# Domain-specific simulated data enhances knife-mark noise suppression in microscopy images of materials

**DOI:** 10.1093/jmicro/dfaf049

**Published:** 2025-10-24

**Authors:** Masato Suzuki, Yasuhiko Igarashi

**Affiliations:** Institute of Engineering, Information and Systems, University of Tsukuba, 1-1-1 Tennodai, Tsukuba 305-8573, Japan; Research & Advanced Development Division Materials Analysis Laboratory, The Yokohama Rubber Co., Ltd, 2-1 Oiwake, Japan; Institute of Engineering, Information and Systems, University of Tsukuba, 1-1-1 Tennodai, Tsukuba 305-8573, Japan; Tsukuba Institute for Advanced Research (TIAR), University of Tsukuba, 1-1-1 Tennodai Tsukuba, Ibaraki 305-8577, Japan; Center for Basic Research on Materials, National Institute for Materials Science, 3-13 Sakura, Tsukuba, Ibaraki 3050003, Japan; Industrial Application and Partnership Division, Japan Synchrotron Radiation Research Institute (JASRI), 1-1-1 Kouto, Sayo-cho, Hyogo 679-5198, Japan

**Keywords:** knife-mark noise, rubber materials, image processing, deep learning

## Abstract

Accurate quantitative analysis of material microstructures from images is often hindered by noise and artifacts generated during sample preparation. While deep learning is a promising approach for this challenge, preparing the large amount of ‘supervised data’ (labeled real images) required for training poses a significant barrier in material science. This study proposes and validates a simulation-driven learning paradigm where a deep learning model is trained exclusively on simulated images that mimic the key features of target structures and noise, serving as a powerful solution to this data scarcity problem. As a specific case study, we applied this paradigm to the removal of ‘knife-mark noise’ from cross-sectional images of rubber materials to enable accurate filler region segmentation. In evaluations using simulated data, the proposed method showed superior performance across all the metrics (PSNR, SSIM, and MAE) compared with conventional methods such as the median filter and TV reconstruction, as well as a U-Net model trained on general-purpose Gaussian noise. More importantly, the model also performed effectively on real images, despite being trained solely on simulated data. It successfully removed both knife-marks and material-derived background textures, which demonstrates the viability of simulation-driven learning to overcome the need for manually annotated datasets. This work highlights the power of task-specific simulations as a practical alternative to manual data annotation in quantitative materials analysis.

## Graphical abstract

**Graphical Abstract:**
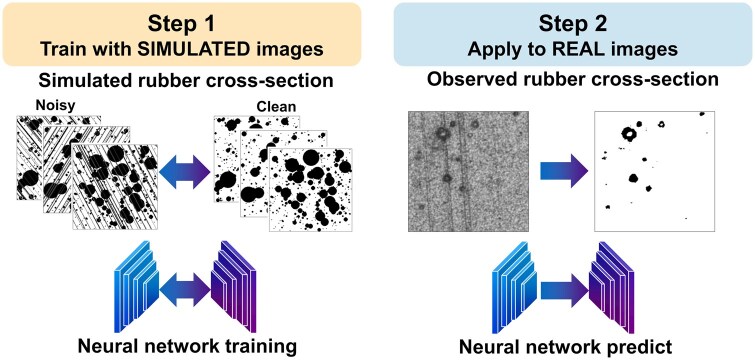

## Teaser

Knife-mark noise often arises in microscopy of materials. Leveraging their simple textures relative to natural images, we simulate knife-marked micrographs and train a deep network without labeled real data. The resulting model surpasses conventional methods, removing artifacts while preserving structure, demonstrating simulation-driven learning as a practical materials-science solution in research.

## Introduction

In materials science, image data obtained using techniques such as atomic force microscopy (AFM), electron microscopy (EM), and optical microscopy (OM) are crucial for analyzing the microstructure and properties of materials [1–9]. The image data obtained through these microscopy techniques enable the visualization of the structure and morphology of materials from the nanoscale to the macroscale without being restricted to specific observation targets. For example, OM has been used to observe the dispersion state of plant-derived biomass waste filler in polyethylene-based composite materials [10], and EM has been applied to observe the structure of graphene [11] and organic molecules on graphene [12] at the atomic level.

However, issues such as reduced analytical accuracy originating from the observation process itself and the introduction of subjectivity or arbitrariness due to manual measurements have also been documented [13–16]. Consequently, many efforts are underway to overcome or ameliorate these problems through image analysis technology [17–21].

Since the nature of noise varies depending on the material, standardized noise reduction techniques typically used for general photographic image analysis are often difficult to apply directly. Therefore, image processing methods must be optimized on the basis of the specific characteristics of each material and task. Previous studies have demonstrated the remarkable potential of deep learning methods for segmentation in materials microscopy images [22–25]. A common challenge in applying these powerful methods, however, is their reliance on manually annotated datasets, which are often resource intensive.

Therefore, this study proposes and validates a new training paradigm for deep learning-based image analysis that bypasses the need for real labeled data. Our approach involves training a model exclusively on simplified, purpose-built simulated images that mimic the key features of the target microstructure and characteristic noise. Leveraging their simple textures relative to natural images, we simulate knife-marked micrographs and train a deep network without labeled real data. We demonstrate that this simulation-based strategy is a powerful and practical solution to the persistent challenge of data scarcity in materials science. To validate the effectiveness and practical viability of this simulation-based paradigm, we apply it to the challenging problem of ‘knife-mark noise’ generated during the sample preparation of polymeric materials.

In the observation of material images, noise can arise from various factors. However, noise generated during the sample preparation process, particularly during material sectioning, is a significant concern, as it critically impacts the reliability of the analysis results and therefore cannot be overlooked. Observing the internal structure of a material often requires sectioning the material to expose a cross-section for examination. This process is essential for the direct evaluation of the internal microstructure and compositional distribution of a material or for the use of observation techniques that require thin samples [26, 27]. However, this sectioning process itself can introduce unintended artifacts into the image. In such instances, stripe-like distortions, known as knife-mark noise, may be introduced. Such noise occurs when, during cross-section preparation using methods such as mechanical cutting, traces from the cutting tool’s edge or from snagging remain on the sample surface as periodic indentations or layers, manifesting as streak-like noise in the observed image [28–31].

This issue is particularly relevant in polymeric materials such as rubber, where the segmentation of filler regions is crucial for accurately understanding the filler distribution, which in turn affects the physical properties of the material [32, 33]. Poor particle dispersion appears as large agglomerates in materials microscopy images. For example, poor filler dispersion has been reported to decrease key mechanical properties, such as tensile strength and elongation at break [34–36]. The analysis of material cross-sections is often complicated by the presence of knife marks [32, 37, 38]. These materials tend to be prone to the formation of irregular knife marks due to the combined effects of multiple factors, such as knife wear and irregular compression between the knife and the material [32, 38]. The difficulty of image analysis increases significantly due to the combined presence of irregular knife marks and background-derived noise [32, 37]. Many conventional image processing methods, particularly those used for stripe noise removal, assume the presence of regular noise patterns in the vertical and horizontal directions [39–42]. Consequently, these approaches are often unsuitable for addressing knife-mark-like noise that exhibits significant variations in angle, as observed in the cross-sections of actual materials. Therefore, there is a need to develop new processing methods capable of effectively handling such complex and irregular image artifacts. In this paper, we first detail the methodology for generating the purpose-built simulated dataset used for training. We then describe the U-Net [23, 43] architecture and the training process. Finally, we present a comprehensive evaluation of the model’s performance on both simulated and real images, comparing it against conventional methods to confirm the effectiveness of our proposed simulation-driven learning paradigm.

## Materials and sample preparation

The real images used in this study were obtained from a rubber sample prepared in-house. The formulation was based on styrene-butadiene rubber (SBR) with the following composition reported in parts per hundred rubber (phr): SBR, 100; HAF-grade carbon black, 61; zinc oxide, 3; sulfur, 1.4; and the vulcanization accelerators N-cyclohexyl-2-benzothiazole-2-sulfenamide (1.7 phr) and 1,3-diphenylguanidine (1.5 phr). All chemicals were commercially available and used as received without further purification. The compounded material underwent press-curing at 160 °C for 20 min to produce a vulcanized rubber sheet.

### Image acquisition

A piece of the vulcanized rubber, approximately 3 cm × 3 cm with a thickness of 2–3 mm, was prepared. A cross-section of this sample was observed using an optical microscope at 100x magnification to acquire digital images. The images were captured at a resolution corresponding to approximately 0.8 µm per pixel. For the purpose of this study, these acquired images were then cropped to a resolution of 256 × 256 pixels to evaluate the results of the image processing. In addition, to validate the practical effectiveness of the methods, we constructed a new test set with manual annotations. Twelve real microscopy images were prepared under two distinct conditions: six obtained from cross-sections cut with a pristine blade (Fig. S1) and six others obtained after deliberately damaging the blade and recutting the sample (Fig. S2). This procedure yielded a diverse set of test images exhibiting varying degrees of knife-mark severity. Importantly, this dataset was used exclusively for testing and was not seen during training.

### Method

In this work, we propose a novel method for precisely extracting filler regions by removing the irregular knife-mark noise present in cross-sectional optical microscope images of rubber materials. This approach is based on constructing a dataset of simulated image pairs. Each pair consists of a ‘ground truth’ image, which represents a noise-free filler dispersion state, and a corresponding ‘noisy image’ created by superimposing simulated knife-mark noise onto the ground truth image. This methodology aims to overcome the challenge of limited real datasets in materials science and achieve robust noise removal performance against diverse noise patterns with varying thicknesses and angles. In this section, we first introduce the generation method of the simulation dataset, followed by an explanation of the proposed U-Net-based noise removal algorithm and the existing methods used for comparative analysis.

### Simulated dataset generation

As discussed in the Introduction, irregular knife-mark noise with varying angles and line thicknesses occurs in material cross-sectional images [28–31]. This characteristic is introduced by the material-sectioning process itself and is not observed in typical general signal-derived stripe noise [39, 40, 44]. On the other hand, when fillers are well dispersed, they exist as primary units (aggregates) on the nanometer to micrometer scale, requiring electron microscopy (e.g. TEM, SEM) for observation [36, 45, 46]. In cases of poor dispersion, however, these units form much larger agglomerates that become detectable by optical microscopy [32, 47]. This degree of dispersion failure is a crucial parameter related to material strength [34–36, 47]; therefore, this task requires appropriate modeling of both these agglomerates and the noise. Given the aforementioned complexities and the inherent challenge of insufficient real data in materials science, we constructed a comprehensive simulation dataset. This dataset aims to enable objective evaluation of noise removal performance under controlled conditions and to overcome the scarcity of real data for model training. In this study, the image resolution was standardized to 256 × 256 pixels. However, the applicability of the proposed method is not limited by this specific resolution. Because the power law used to generate the simulated fillers has scale-free characteristics, the method remains theoretically valid for images of different resolutions, provided that they do not contain particles exceeding the maximum diameter defined during the simulation.

**Fig. 1.**
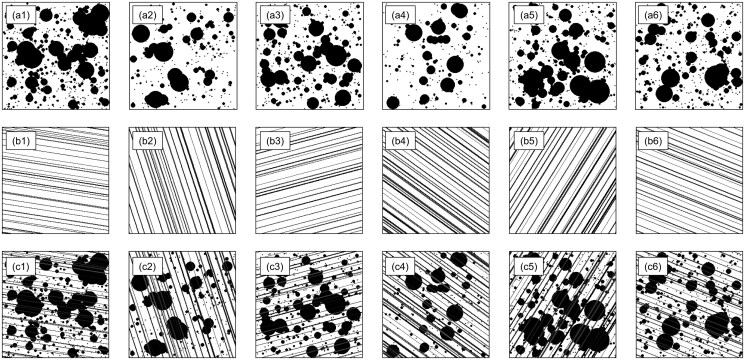
Representative examples of the constituent elements of the dataset generated via simulation. The dataset was generated to train and evaluate the noise-removal model and consists of images with a resolution of 256 × 256 pixels. (a) Ground truth image showing a noise-free, ideal filler particle dispersion state. The particle radii were simulated to follow an inverse power-law distribution. (b) Noise pattern images generated by simulating knife-mark noise that is introduced during sample preparation. (c) Noisy images, which serve as input for the machine learning model, created by superimposing the knife-mark noise (b) onto the ground truth images (a).

Figure 1 presents representative examples of the types of images that constitute the simulation dataset constructed using the aforementioned method. The figure visualizes the fundamental components of the image pairs used for training and evaluating the noise removal methods proposed in this study. First, Fig. 1a is defined as the ‘ground truth image’, representing the ideal dispersion state of filler particles, which is completely unaffected by noise. Since the purpose of this task is the detection of filler agglomerates and the assessment of their dispersion state, the simulation in Fig. 1a is designed by placing spherical structures that exclusively simulate these filler agglomerates. The probability of a particle’s radius appearing is designed to follow an inverse power law. To ensure the simulated particles were representative of the experimental samples, the maximum agglomerate radius in the actual rubber images was first measured (19 pixels), and the maximum radius for simulated particles was accordingly set to 28.5 pixels (19 × 1.5). The detailed methodology for generating these simulated filler images is described in the Supplementary Material (Fig. S5). This power law distribution is scale-free, allowing for the generation of images that maintain representativeness across various observation magnifications. Furthermore, its prevalence in natural phenomena [48–50] and material particles [51, 52] ensures that the simulated particle sizes reflect the diverse and universal distributions found in real materials. This ground truth image is the target state that the machine learning model should ultimately restore, and it functions as the correct data for the noise removal process. To confirm the statistical validity and uniformity of the generated images, we conducted a convergence analysis of the particle area distribution. The results, as detailed in the Supplementary Material (Figs S3 and S4), demonstrate that our simulation produces a uniform and reproducible particle distribution consistent with its theoretical design.

Figure 1b shows an example of a noise pattern generated by simulating the ‘knife-mark noise’ that occurs due to a combination of factors, such as knife degradation or interaction with carbon black particles, during the preparation of cross-sectional samples of soft composite materials such as rubber. This noise pattern is designed to continuously reproduce the irregular and diverse knife-mark noise frequently encountered during actual rubber material observation, which has been challenging to address with conventional removal techniques. This approach aims to facilitate the development of a more practical noise removal model. The detailed methodology for generating this simulated knife-mark noise is described in the Supplementary Material (Fig. S6). This noise is modeled by drawing multiple straight lines with widths of 1–3 pixels at a random angle θ for each image to reproduce the irregularities observed in actual material images. The brightness of these linear noises is also randomly set in the 0 (dark color), similar to the filler particles, and intentionally includes irregular diagonal patterns and variations in brightness and thickness that are difficult to address with conventional stripe noise processing methods.

Finally, Fig. 1c is a ‘noisy image’ created by superimposing the simulated knife-mark noise (Fig. 1b) onto the ground truth image (Fig. 1a). This noisy image is intended to provide the model with training data that pair known filler structures (Fig. 1a) with superimposed realistic noise (Fig. 1b). This approach enables the model to efficiently learn noise removal patterns even when acquiring annotated real data is difficult. This noisy image is input into deep learning models such as U-Net, which was developed in this study, and the model learns to extract the filler regions contained in the original ground truth image accurately (Fig. 1a) or to reconstruct the ground truth image itself by removing noise components. In this work, a total of 1000 pairs of ground truth images and corresponding noisy images were generated and used as a dataset for performance evaluation. This approach addresses the shortage of actual image datasets, which is a particular challenge in the field of materials science, and establishes a foundation for objectively evaluating the performance of noise removal models under controlled conditions.

### Proposed method for knife-mark noise removal using U-Net

U-Net is a deep learning model originally developed for the segmentation of biomedical images [43]. Its characteristic U-shaped architecture features pathways that connect the contracting path (encoder), which captures context, with the expansive path (decoder), which enables precise localization. This design facilitates high-precision segmentation and has since been applied to a wide range of image processing tasks beyond its original biomedical domain [53–59]. In this study, we adapted the U-Net model to remove knife-mark noise from cross-sectional images of rubber materials by training it with a simulated dataset. Specifically, the model was trained using a noisy image with superimposed knife-mark artifacts as the input and the corresponding noise-free image representing the ideal filler dispersion state as the ground truth target. By learning from this paired dataset, the U-Net model was configured to selectively remove noise components from the input and accurately extract, or segment, the filler regions. The model was trained using a dataset of 4000 simulated image pairs, which were generated via simulation under the same conditions as the 1000-pair dataset used for performance evaluation. This dataset was partitioned into training and validation sets at a 7:3 ratio. The training methodology was specifically designed to account for the physical nature of the cross-sectioned samples. In the observed images, filler agglomerates are not always perfectly exposed on the surface but are often embedded at varying depths within the rubber matrix, resulting in perceptual ambiguity. Agglomerates located deeper beneath the surface appear as fainter, less distinct regions, making them difficult to distinguish from noise, even for a human observer. To model this inherent ambiguity, we employed a ‘soft labelling’ approach. Instead of converting the ground truth image to a rigid binary mask (filler/not filler), we used the grayscale image directly as the training target. The pixel intensity values, normalized to the [0, 1] range, serve as soft labels. In this scheme, a value closer to 0 represents a clearly exposed filler, a value closer to 1 represents the background, and intermediate values correspond to these ambiguous subsurface features. Consequently, the U-Net model was trained using binary cross-entropy loss to output a probability map that reflects this degree of confidence. This approach leverages binary cross-entropy not for strict binary classification but to enable the model to predict the continuous confidence level of a pixel belonging to a filler, mimicking the expert observer’s nuanced judgment of features embedded at varying depths. This method enables the network to learn not only the location of fillers but also the uncertainty associated with features embedded within the material. The model was optimized using the adaptive moment estimation (Adam) algorithm with an exponential decay learning rate schedule, starting from an initial rate of 0.0001. The training was conducted for 60 epochs with a batch size of 16. The model was trained on a workstation equipped with an Intel Core i9-14900K CPU, 64 GB of RAM, and an NVIDIA RTX 4500 Ada Generation GPU. To ensure reproducibility, additional details are summarized here. Both the proposed U-Net (knife-mark noise) and the comparative U-Net (Gaussian noise) used the same hyperparameter settings for a fair comparison. The final layer employed a sigmoid activation function to output probability maps, and the learning rate decayed by a factor of 0.9 every 100 steps. As shown in Fig. S7, both models exhibited smooth convergence, with validation losses closely following the training losses throughout the process, confirming that the training was stable and free from overfitting.

### Comparison methods for noise removal algorithm

To comprehensively evaluate the performance of the proposed noise removal method, we compared it against several established techniques. These comparative methods can be broadly categorized into three main approaches: image filtering, statistical machine learning, and deep learning-based methods. This section details each of these approaches.

### Filtering approaches

Median Filter: The median filter was selected from conventional image processing techniques to serve as a comparative method [60]. This filter operates by outputting the median pixel value within a local image region, which makes it effective at smoothing fine linear structures and minute, point-like noise. Owing to this characteristic, the median filter is sometimes utilized as a component in methods designed for removing vertical stripe noise [61].

Band stop Filter: A band stop filter that operates in the frequency domain was selected. This filter is effective at removing periodic noise by transforming an image into the frequency domain via the fast Fourier transform (FFT), removing components within a specific frequency band, and then transforming it back to the spatial domain using the inverse fast Fourier transform (IFFT). Since the knife-mark noise targeted in this study characteristically occurs irregularly and at various angles, an annular (ring-shaped) mask was adopted to isotropically remove frequency components without being limited to a specific orientation. Such filtering in the frequency domain is generally used as a removal method when the noise exhibits strong periodicity [62, 63]. As knife marks possess a periodic structure, their frequency components are localized in the spectrum; therefore, this method is considered effective for their removal.

### Statistical machine learning approaches

#### Noise removal via image reconstruction (L1 and TV regularization)

As comparative methods based on sparse modeling and image reconstruction, we selected L1 regularization and total variation (TV) regularization reconstruction for noise removal [64–71]. These techniques were introduced to reconstruct a denoised image by minimizing a regularization term while maintaining fidelity to the original noisy image. The key parameter in this process is the regularization coefficient, λ, which controls the balance between these two objectives. L1 regularization is a method that minimizes the L1 norm, defined as the sum of the absolute values of the pixel intensities across the image. This approach is based on the assumption that the principal components of the image are sparse. In this study, the task is to extract filler regions that occupy a relatively small area from a large background. L1 regularization is well suited for reconstructing such sparse signals and is therefore considered effective for this task. TV regularization minimizes the total variation in an image, which is the sum of the magnitudes of the intensity gradients between adjacent pixels. This process suppresses abrupt changes in intensity, resulting in a smoothed reconstruction. The knife-mark noise targeted in this study manifests as sharp intensity variations that span the entire image; therefore, the smoothing provided by TV regularization is considered effective for removing this type of noise.

General Stripe Removal (GSR): We also selected the general stripe removal (GSR) developed by Rottmayer *et al.* [42]. This method, an extension of the work of Liu *et al.* [72], is designed to remove stripe artifacts from various 2D and 3D images, such as those from light-sheet fluorescence microscopy (LSFM), electron microscopy, and remote sensing. The GSR is an image reconstruction-based method that incorporates a regularization term to minimize image variation in the direction perpendicular to a specified stripe orientation. This technique requires the orientation of the target noise to be provided as an input parameter. Since the knife-mark noise in this study occurs irregularly and at various angles, GSR, which presupposes a single angle input, is not necessarily an optimal approach for this task. Nevertheless, it was included as a comparative method to evaluate its performance as a representative, general-purpose stripe removal technique.

### Deep learning-based approaches

U-Net (Gaussian Noise): To confirm the importance of training a model specialized for a specific noise type, a U-Net model trained based on Gaussian noise was prepared as a comparative baseline. The noisy images for this comparative baseline were created by adding Gaussian noise with a mean of 0 and a standard deviation of 100 to the ground truth images. In deep learning methods for images, one approach is to train models using simulated images for application to real data [73–75], which is also leveraged in microscopy images [76–81]. For example, in a study by Rahman *et al.* [82], noise following a uniform distribution was used to reproduce background texture in an SEM image segmentation task. In contrast, this study accounts for the complexity and randomness of the background originating from the rubber material’s cross-section and thus selects Gaussian noise as a more general, nonstructural noise model. The U-Net architecture of this comparative model, as well as all of its training parameters, such as the optimizer and learning rate, were identical to those of the proposed method. The only difference was the training data: images created by adding Gaussian noise, instead of knife-mark noise, to the ground truth images were used as input. This design allows for an evaluation of whether training based on structural noise (knife-marks) versus nonstructural noise (Gaussian) is more effective for accomplishing the task.

### Evaluation metrics and experimental setup

In this study, all the captured and simulated images were 8-bit grayscale images. The performance metrics from the 1000 test images are presented as violin plots to show the full distribution of the results (Fig. 3). All data processing and metric calculations were performed using custom scripts in Python 3.9. The pixel values were denoted as $I_{\left(x,y\right)},\;$where the horizontal coordinate x is defined as x∈{0,…,M−1} and the vertical coordinate *y* is defined as y∈{0,…,N−1}. The image size is M×N pixels, and to ensure consistency in the experimental conditions, M=N=256 was set in this study. For evaluation, we utilized high-quality reference images, referred to as ‘ground truth’ images $I_{GT}$, and output images $I_{\mathrm{out}}$ obtained after applying image processing and Otsu’s binarization [83] to noisy images.

In this study, we employed multiple objective evaluation metrics to quantitatively assess the quality of generated or processed images. The primary metrics used were the peak signal-to-noise ratio (PSNR), structural similarity index (SSIM), and mean absolute error (MAE). The PSNR measures the pixel-level fidelity between the ground truth ($I_{GT}$) and output images ($I_{\mathrm{out}}$), with higher values indicating better reconstruction quality. $I_{\max}$ is the maximum pixel value, and the mean squared error (MSE) is defined below.


$$M\begin{array}{c} SE=\frac{1}{MN}\sum_{x=0}^{M}\sum_{y=0}^{N}{\left|I_{GT(x,y)}-I_{\mathrm{out}(x,y)}\right|}^{2}\\ \mathrm{PSNR}=10\log_{10}\frac{I_{\max}^{2}}{\mathrm{MSE}} \end{array}$$

The SSIM measures structural similarity using luminance, contrast, and structure, aiming for an evaluation that is more aligned with human visual perception than the PSNR is.


$$\mathrm{SSIM}=\frac{\left(2\mu_{x}\mu_{y}+C_{1})(2\sigma_{xy}+C_{2}\right)}{\left(\mu_{x}^{2}+\mu_{y}^{2}+C_{1})(\sigma_{x}^{2}+\sigma_{y}^{2}+C_{2}\right)}$$

where μ is the mean intensity, σ is the variance, $\sigma_{xy}$ is the covariance, and $C_{1}$ and $C_{2}$ are constants introduced to prevent division by zero. The local means and variances were calculated using a 7 ×7 pixel sliding window.

The MAE calculates the average absolute difference between the corresponding pixel values in the original and processed images.


$$\mathrm{MAE}=\frac{1}{MN}\sum_{x=0}^{M}\sum_{y=0}^{N}\left|I_{GT(x,y)}-I_{\mathrm{out}(x,y)}\right|$$

In the ‘Quantitative Evaluation Based on a Large-Scale Simulation Dataset’ section, parameter searches were conducted with the objective of minimizing the MAE using the simulated validation dataset. For sparse modeling-based methods (L1 regularization and TV regularization), we searched for the regularization parameter λ within the range of 0–3. The detailed hyperparameters for all the conventional methods (e.g. kernel size for the median filter and angle for the GSR) and the full specifications of the U-Net models, including architecture and training parameters, are described in the Supplementary Material (Section S6). For the real-image evaluation (Application to Real Images and Validation of Effectiveness), we first created ground‑truth masks by manually annotating real images. We then optimized the hyperparameters of each conventional method to minimize MAE (the average of 12 images) with respect to these annotations. An exception was made for GSR. When we attempted to minimize MAE directly on the real images, the strong background texture—rather than the stripe artifact—dominated the objective, leading to ineffective angle selection. We therefore used an angle parameter that had been confirmed to be effective for removing linear artifacts in the simulation dataset. In contrast, the proposed U‑Net model—trained solely on simulated data—was applied to the real images without any fine‑tuning or real‑image‑specific parameter adjustment, in order to rigorously assess its generalization performance. The detailed parameter settings are provided in Sections S5 and S6 (see also Figs S9 and S10).

## Results and discussion

### Performance evaluation of noise removal based on simulated data

**Fig. 2.**
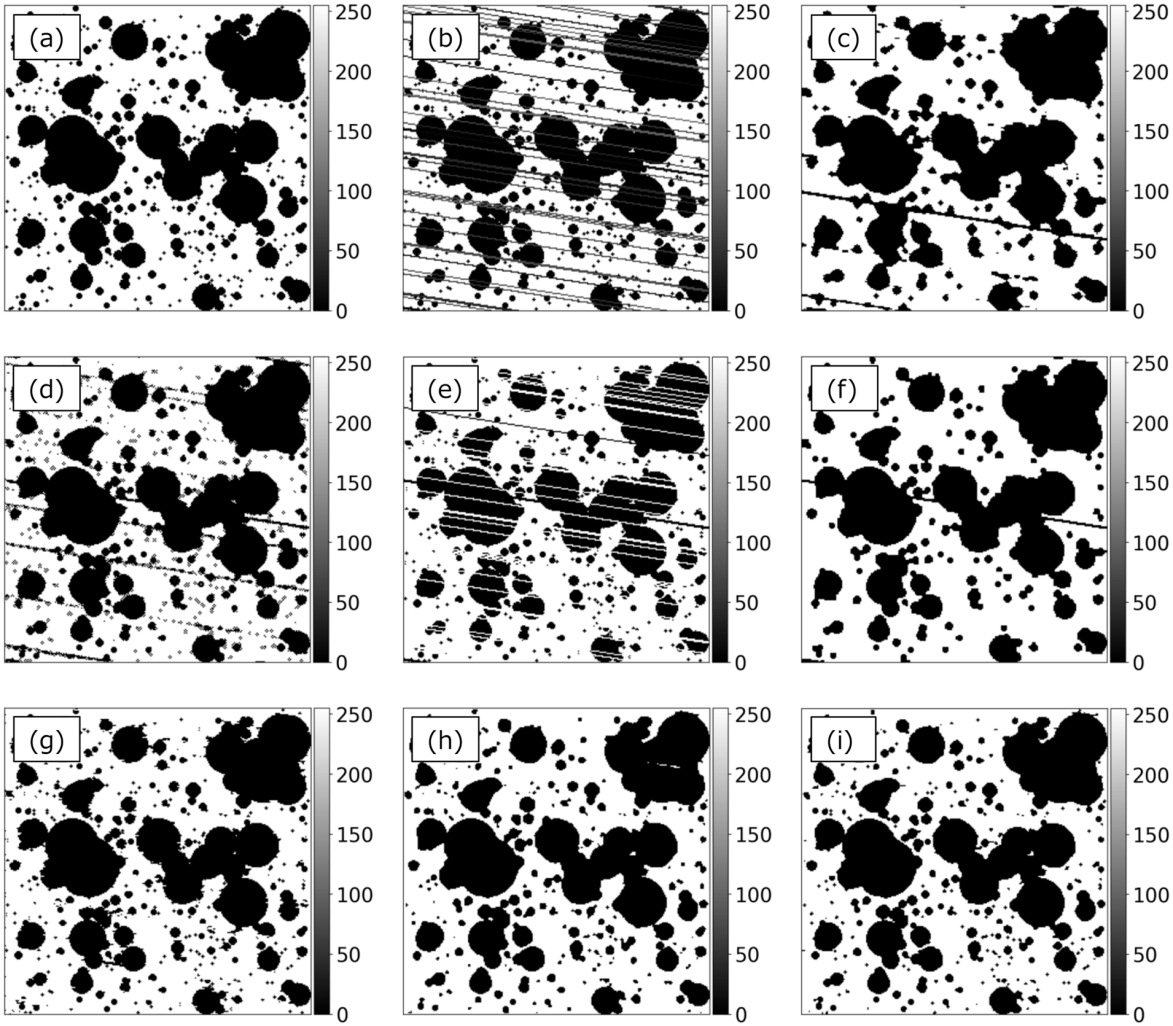
Visual comparison of the noise removal results for a representative simulated image. (a) ground truth, (b) noisy image, (c) median filter, (d) band stop filter, (e) L1 reconstruction, (f) TV (total variation) reconstruction, (g) general stripe removal, (h) U-Net (Gaussian noise) and (i) the U-Net model (knife-mark noise).

First, to establish a baseline for performance, the parameters of conventional methods were optimized, and a qualitative comparison with the proposed method was conducted using a representative simulated image. A qualitative comparison of various noise removal methods was conducted using a representative simulated image containing knife-mark noise, and the results are presented in Fig. 2. To facilitate a clear comparison of filler region extraction accuracy, all processed images were binarized using Otsu’s method [83]. For each method, the relevant parameters (such as the filter size, regularization coefficient, and angle) were optimized by minimizing the MAE against the ground truth image. The median filter [60] (Fig. 2c), a conventional spatial filtering technique, provided some degree of noise reduction; however, thick linear artifacts persisted in the resulting image. Compared with the median filter, the band stop filter [62, 63] (Fig. 2d), which operates in the frequency domain, was more effective at preserving fine particles; however, its removal of linear noise was also incomplete.

Among the image reconstruction-based methods, L1 reconstruction [71] (Fig. 2e) was able to suppress some of the noise lines but also caused oversuppression of areas where fillers and noise overlapped, introducing new white artifacts. In contrast, TV reconstruction [70] (Fig. 2f) was successful in removing many of the lines but also led to the unintended disappearance of some fine particles. GSR [42] (Fig. 2g), when applied with an angle optimized to minimize the MAE, selectively removed a significant portion of the noise and demonstrated high performance among the conventional methods. The U-Net model (Gaussian noise) (Fig. 2h), which was trained with general-purpose Gaussian noise, was capable of noise removal; however, it introduced some partial distortions to the filler structures. In contrast, the proposed method, the U-Net model (knife-mark noise) (Fig. 2i), was the most effective at removing the knife-mark noise while simultaneously and consistently preserving the shape and distribution of the particles. Consequently, it yielded the result that most closely resembled the ground truth data (Fig. 2a). This visual comparison demonstrates that constructing a deep learning model with simulated images that mimic both knife-mark noise and filler morphology is a highly effective strategy for noise removal (see also Fig. S8 for the quantitative comparison of MAE values). This result indicates that the convolutional architecture of the U-Net is particularly well-suited for addressing structured, large-scale noise, such as the knife-marks that span the entire image.

To support the visual evaluation, we quantitatively assessed the performance of each method using the MAE. A lower MAE signifies more faithful noise reduction to the ground truth image. The proposed U-Net model (knife-mark noise) achieved a minimum average MAE of 3.27 [1.28% of the full 8-bit pixel range (255)], demonstrating the highest quantitative performance (Fig. 2i). Among the conventional methods that do not require training, GSR performed well, with an MAE of 3.79 (1.49%) (Fig. 2g). However, the proposed method outperformed the GSR method. Furthermore, the MAE of the U-Net trained with general-purpose Gaussian noise was 6.15 (2.41%) (Fig. 2h), confirming the importance of training with task-specific noise. Although our evaluation is limited to a single representative image, we verified that the proposed method delivers visual quality that matches, and in some cases surpasses, that of conventional approaches individually tuned for the same image.

### Quantitative evaluation based on a large-scale simulation dataset

**Fig. 3.**
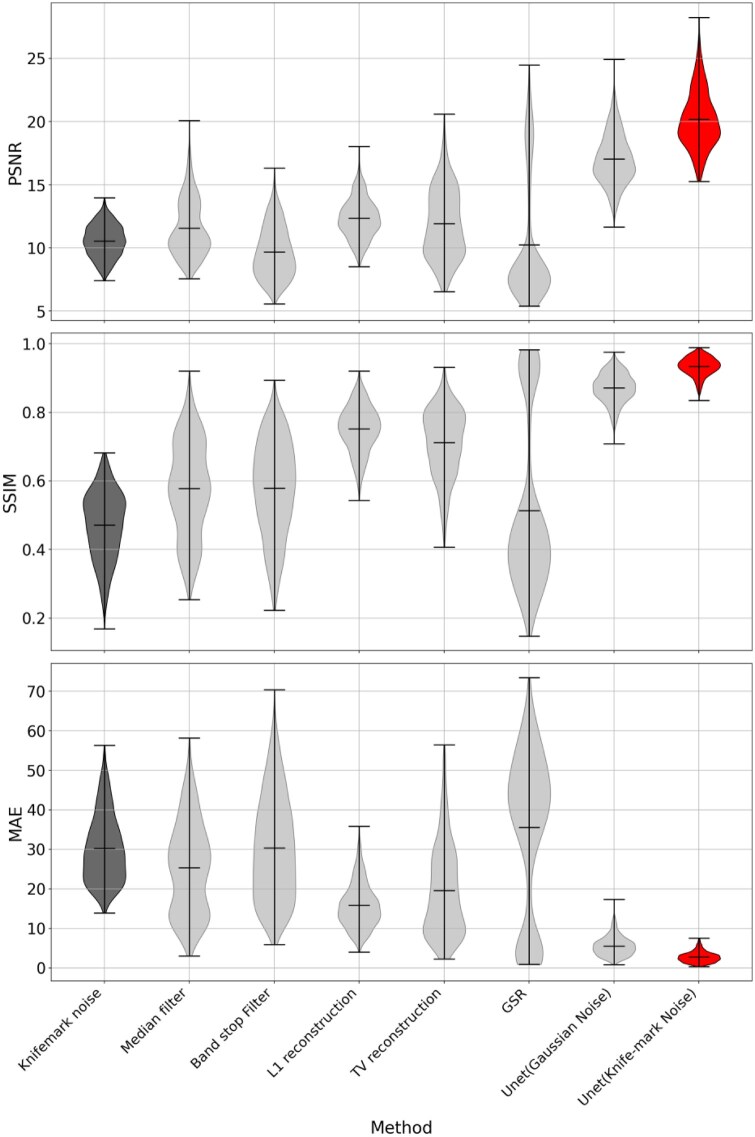
Quantitative evaluation of noise removal methods using a large-scale dataset of 1000 simulated images. The violin plots show the distribution of performance for each method across three metrics: the peak signal-to-noise ratio (PSNR), structural similarity index (SSIM), and mean absolute error (MAE). Higher PSNR/SSIM values and lower MAE values indicate better performance. The ‘Knife-mark noise’ violin plot represents the performance of the original noisy images before any noise removal process was applied, serving as a baseline.

A large-scale quantitative evaluation was conducted to assess the performance and stability of the methods more rigorously. For this purpose, a simulation dataset of 1000 pairs was used. The parameters for each method were fixed to the values optimized in the previous section. The distributions of the results for the evaluation metrics—PSNR, SSIM, and MAE—are shown as violin plots in Fig. 3. First, we considered conventional, general-purpose methods (median filter, band stop filter, L1 reconstruction, TV reconstruction). For all of these methods, the distribution of the SSIM, in terms of both median and mean values, shifted toward 1.0 compared with that using the unprocessed image (Knife-mark noise). This finding suggests a certain degree of effectiveness in noise removal from the perspective of structural similarity. However, for the band stop filter, numerous cases were observed in the PSNR and MAE distributions, where the performance was worse than that of the unprocessed image. This finding indicates that, depending on the frequency characteristics of the image, the separation of noise and filler components can become difficult, leading to a significant degradation in performance. Among these four methods, L1 reconstruction tended to exhibit the narrowest distribution width (variance) for all the metrics. This outcome is likely attributable to the fact that L1 reconstruction targets the luminance values themselves rather than the image structure, resulting in relatively high robustness against the geometric patterns of the noise.

The GSR exhibited a bimodal distribution across all metrics, underscoring both its primary strength and its main limitation in automated workflows. Owing to its high sensitivity to the input orientation, GSR can achieve near‑perfect noise removal when the specified angle matches the stripe artifact, with many data points showing SSIM close to 1.0 and MAE close to 0. This indicates that, under the correct angle, GSR can surpass the proposed method in specific cases. However, this reliance on a correct angle parameter makes automation difficult for real‑world images with complex characteristics, such as strong background–stripe blending or curved/locally varying stripes (Fig. S12), for which a single global angle is ill‑defined. Therefore, while GSR may be less suitable for fully automated pipelines, it remains an extremely powerful option in scenarios where an operator can manually identify and set the optimal removal angle. Finally, a comparison is made between the two deep learning-based methods. Compared with those of U-Net trained with general-purpose Gaussian noise, the proposed U-Net model (knife-mark noise) clearly yields superior mean values (the central black line in the distribution) across all the metrics, ie, the PSNR, SSIM, and MAE. Furthermore, the proposed method yields narrower distribution widths for the MAE and SSIM than any other methods do, confirming that its performance has extremely low variance. This finding indicates that by training specifically for the task of knife-mark noise removal, the proposed method can extract filler regions with high stability and accuracy against various noise patterns. From these results, it can be concluded that the proposed method has the best overall noise removal performance among all the compared techniques in terms of both its high average performance and its stability (robustness) against diverse image patterns.

### Application to real images and validation of effectiveness

**Fig. 4.**
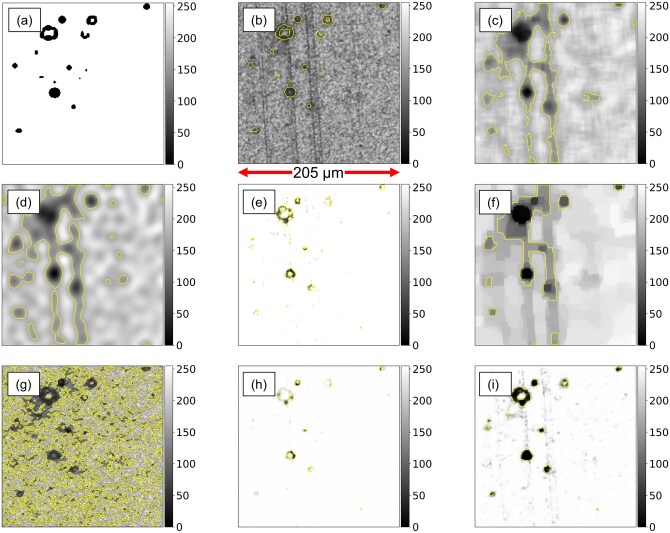
Comparison of various noise removal methods applied to a real microscopy image (representative example from the 12 annotated test images). Contours of the extracted filler regions (obtained after binarization using Otsu’s method) are overlaid on each panel. (a) ground truth, (b) noisy image, (c) median filter, (d) band stop filter, (e) L1 reconstruction, (f) TV (total variation) reconstruction, (g) general stripe removal (GSR), (h) U-Net (Gaussian noise) and (i) U-Net (knife-mark noise).

**Fig. 5.**
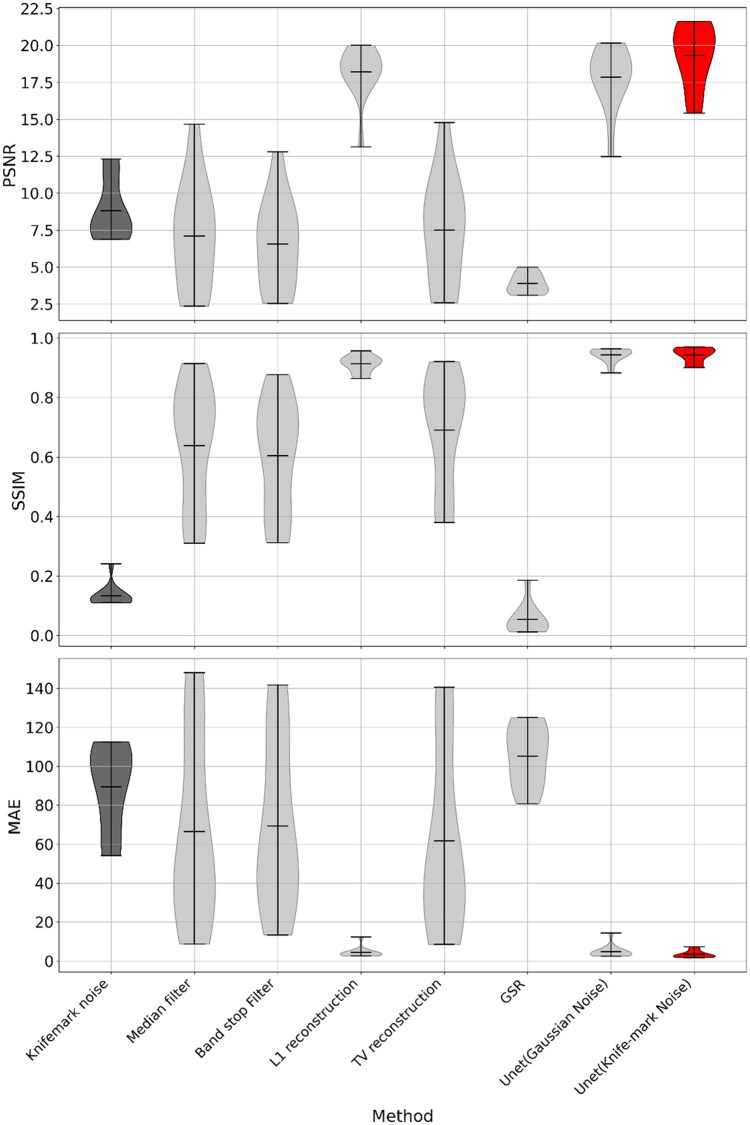
Quantitative evaluation of noise removal methods using 12 manually annotated real images. The violin plots show the distributions of PSNR, SSIM, and MAE for each method. Conventional methods (median, band stop, TV) exhibited wide errors and occasional severe degradation (e.g. MAE > 120). The proposed U-Net (knife-mark noise) achieved the highest PSNR (19.33), SSIM (0.94), and the lowest MAE (3.34), demonstrating superior accuracy and stability.

To validate the effectiveness of each method using real images, the methods previously confirmed using annotated data were applied to an actual image of a rubber material cross section. Figure 4 provides a visual comparison of the extracted filler regions after binarizing the processed images using Otsu’s method. This real image contains not only the primary target of knife-mark noise but also a fine background texture derived from the rubber itself, which is difficult to account for in simulations; therefore, it serves as a critical test for the robustness of each method. The distributions of the results for the evaluation metrics—PSNR, SSIM, and MAE—are shown as violin plots in Fig. 5. When the conventional methods were applied, the median filter (Fig. 4c) partially smoothed the fine background texture but failed to remove the primary knife-mark noise, which remained in the image. Quantitatively on the annotated real‑image test set (12 images), the median filter achieved mean PSNR 7.11, SSIM 0.64, and MAE 66.45 on average, with a wide dispersion in the violin plots (Fig. 5). With the band stop filter (Fig. 4d), both the knife marks and the background texture were detected as noise, leading to incorrect extraction of the filler regions. This behavior is reflected in the real-image metrics (PSNR 6.57, SSIM 0.61, MAE 69.32) and the broad spread of the violin plots (Fig. 5). L1 reconstruction (Fig. 4e) is an approach that focuses on removing noise by assuming that it is a sparse signal and minimizing the L1 norm of luminance values. L1 reconstruction performed strongly in pixel-wise fidelity (PSNR 18.20, SSIM 0.913, MAE 4.27). While this method produces promising results overall, its weaknesses become more apparent when compared with our proposed method: in regions where filler and noise intensities overlap, extraction of the filler regions remains incomplete. In contrast, TV reconstruction (Fig. 4f) is a method that smooths the image by minimizing the sum of the luminance gradients of the entire image (total variation). This characteristic caused excessive smoothing of boundaries between filler regions that should have remained separate, leading to their unnatural connection and deformation. Results show that the mean values of PSNR, SSIM, and MAE (7.50, 0.691, and 61.68, respectively) exhibited a long-tailed MAE distribution. When the GSR method was applied with a correctly matched angle (as shown in Fig. S11), it cleanly removed the linear stripe artifacts. However, a significant amount of background noise originating from the material itself remained. This residual texture interferes with downstream tasks such as region extraction via binarization (Fig. 4g), with mean values of PSNR = 3.89, SSIM = 0.05, and MAE = 105.14. Therefore, for our specific application, the GSR method would need to be combined with a separate background noise removal technique to accurately segment the filler regions. This highlights the advantage of our proposed method, which addresses both stripe artifacts and background texture in a single process. With respect to the deep learning models, both U-Net (Gaussian noise) (Fig. 4h) and the proposed U-Net (knife-mark noise) (Fig. 4i) models succeeded in effectively removing both the complex knife marks and the background texture unique to the real image, extracting only the filler regions. This finding is notable, as both models were trained solely with simulation data. However, a detailed comparison of the two models revealed that U-Net (Gaussian noise) tended to overremove parts of the filler agglomerates in the upper portion of the image. In contrast, the proposed U-Net (knife-mark noise) (Fig. 4i) model preserved the shape of the filler agglomerates more accurately, thereby reducing false positives (mistaking noise for fillers). Quantitatively, the U-Net (Gaussian noise) achieved mean PSNR = 17.85, SSIM = 0.94, and MAE = 4.80, whereas the proposed U-Net (knife-mark noise) achieved mean PSNR = 19.33, SSIM = 0.94, and MAE = 3.34. In addition, the violin plots in Fig. 5 demonstrate that the proposed model achieves both higher accuracy and greater stability, with narrower error distributions across the test set.

Detailed results for all twelve test images are provided in Section S9 (Figs S13–S24). Despite variations in blur and contrast caused by slight differences in imaging conditions, the proposed model consistently extracted filler regions accurately, demonstrating robustness against image quality variations. Overall, the proposed U-Net consistently outperformed the conventional methods, yielding the highest PSNR and SSIM values and the lowest MAE, thereby confirming its superior generalization to real microscopy images. This result strongly suggests that specializing the training on the structural features of knife-mark noise using simulation data directly contributes to a high generalizability and a more precise filler extraction capability using real image data. These results strongly suggest that the proposed method achieves not only high accuracy and stability for simulation data but also excellent effectiveness for real images. The method is applicable not only to rubber cross-sections but also to various polymer materials, and its expansion to images acquired by other modalities, such as EM and AFM, beyond optical microscopy, is also anticipated. By tailoring the simulated noise to each modality, the framework can potentially address a broader class of problems, enhancing its generalizability. Furthermore, this framework has demonstrated effectiveness not only for structured, highly regular stripe artifacts but also for irregular, non-periodic noise with strong background intensity variations. This feature suggests that the method also has potential applicability to samples such as soft matter [29, 31] and metallic materials [17, 28], where delicate structures are easily scratched or deformed during sectioning.

## Conclusion

By training a deep learning model exclusively on simple simulated images and applying it to the task of extracting filler regions from noisy rubber cross-sections, this study provides a concrete example of how the need for manually annotated datasets—a significant barrier in materials science—can be successfully bypassed. This achievement demonstrates that, particularly in the field of materials science, where securing labeled datasets for training is challenging, an approach that uses simple geometrically simulated images that faithfully replicate real phenomena as a data augmentation strategy is an effective solution for deep learning-based image analysis. Thus, it is possible to train robust and highly accurate models without relying on limited real data.

Compared with that of existing general-purpose image analysis methods, the proposed approach achieves high accuracy. Notably, while conventional stripe noise removal techniques require specific angle designations, this method not only removes knife-mark noise with sufficient accuracy without angle specification but also effectively removes fine background noise originating from the material itself. Furthermore, these findings suggest the importance of selecting appropriate noise models in simulations, demonstrating that modeling task-specific knife-mark noise leads to superior performance compared with that using general Gaussian noise.

This approach can be a highly effective method in a field where training data are limited. However, the effectiveness of this method remains unknown for more complex structures and noise that cannot be modeled with the simple geometric elements used in this study. When dealing with complex subjects, the key to improving performance lies in how closely the simulated images can replicate reality. One future direction is the introduction of higher-fidelity simulations on the basis of physical laws. Research into simulation technology is particularly advanced in the field of EM images [84, 85], and leveraging these techniques is promising. However, physics-based simulations tend to have increasing computational costs as they pursue greater realism, which could make it difficult to generate large-scale datasets, such as the 4000-image set used in this study.

Therefore, as an alternative to costly physics-based simulations, we envision the use of generative adversarial networks (GANs). For example, a model such as CycleGAN, which excels at style transfer between unpaired image domains [86^,^^87^], could be considered. CycleGAN can learn a transformation mapping from separate, unlabeled sets of ‘simple simulated images’ and ‘real images’, making it possible to translate low-cost simulated images into more photorealistic ones. By applying this technology, we plan to advance research into efficiently generating high-precision training data to further improve the model’s versatility and performance.

## Supplementary Material

dfaf049_Supplementary_Data

## Abbreviations

AFM: Atomic force microscopy
EM: Electron microscopy
FFT: Fast Fourier transform
GSR: general stripe removal
IFFT: Inverse fast Fourier transform
MAE: Mean absolute error
MSE: Mean squared error
OM: Optical microscopy
phr: Parts per hundred rubber
PSNR: Peak signal-to-noise ratio
SBR: Styrene-butadiene rubber
SEM: Scanning electron microscopy
SSIM: Structural similarity index
TEM: transmission electron microscopy
TV: Total variation
U-Net: A specific type of convolutional network for biomedical image segmentation
