# Supplementary material for: Domain-specific simulated data enhances knife-mark noise suppression in microscopy images of materials
Source: Microscopy (Oxf). 2025 Oct 24;75(4):318–31. doi: 10.1093/jmicro/dfaf049 (PMC13454694; doi:10.1093/jmicro/dfaf049)
Supplement: Supplementary file 1 [file dfaf049_supplementary_data.docx]

**1. Preparation of Test Images with Varying Knife-Mark Severity**

Figure S1. Normal knife-marked cross-sections (a–f).

Figure S2. Severe knife-marked cross-sections (g–l).

We first cut the sample with an intact blade and captured three cross-sectional images, from which two cropped regions (256×256) were taken from each image (Figure S1). We then deliberately damaged the cutter blade, repeated the cutting, and again captured three cross-sectional images with two cropped regions taken from each (Figure S2). This procedure produced a total of 12 test images representing both normal cuts and severely knife-marked surfaces.

**2. Verification of Simulation Uniformity**

Figure S3. Convergence of area distribution uniformity with an increasing number of simulation images. The horizontal axis represents the number of simulation images, and the vertical axis shows the normalized standard deviation, a metric for distribution uniformity. The data shown is an average of five independent simulation runs. The solid line indicates the mean value across these runs, while the shaded region represents the 1$\boldsymbol{\sigma}$ range (mean ± 1 standard deviation), which contains approximately 68% of the results. This metric is calculated by normalizing the standard deviation of the total area distribution across all particle sizes by its mean; therefore, a value closer to zero signifies a more uniform distribution.

To verify the statistical reliability of our simulation, we evaluated the uniformity of the particle distribution. The simulation is designed such that the frequency of a particle with radius (r) follows an inverse square law (1/r²). Under this law, the number of particles (∝ 1/r²) and the area of a single particle (∝ r²) cancel each other out; therefore, the total area occupied by any particle size group is theoretically expected to be constant. To experimentally demonstrate this theoretical uniformity, we calculated the total area for each particle size (in 1-pixel increments) from the generated images. The variation in this distribution was then quantified as the normalized standard deviation (Coefficient of Variation, CV). This evaluation was performed on a cumulatively growing dataset: first on the initial 100 images, then on the initial 200, and so on, up to the full set of 1,000 images. Furthermore, to ensure the results were reproducible, this entire process was repeated five times.

In every trial, the results showed that the Coefficient of Variation (CV) reproducibly converged to approximately 0.05 or less once 1,000 images were evaluated. This indicates that the variation in the area distribution is merely 5% of the mean value. These results demonstrate that our simulation can generate a uniform particle distribution that is faithful to its theoretical design and exhibits high reproducibility.

Figure S4. Stacked Histogram of Total Area by Particle Size.

This stacked histogram illustrates the total area for each particle radius. The analysis is based on 1,000 synthetic images, which have been grouped into 10 distinct sets of 100 images.

To assess the statistical validity of the simulation output, the particle size distribution was quantitatively analyzed. The total area occupied by each discrete particle size class (1-pixel increments) was calculated and found to be consistent across the entire particle size range. This result confirms that the simulation successfully generates images with a uniform particle area distribution, as per its design.

**3. Ground Truth Image Generation Algorithm**

 The Ground Truth images (noise-free images of filler dispersion states) used in this study were programmatically generated according to the following procedure. The image size was standardized to a width and height of 256 pixels. The images were set as 8-bit grayscale, with a white background (brightness value of 255). The proportion of the total area occupied by fillers (coverage ratio) was randomly varied for each image. Specifically, for each generated image, a target coverage ratio was randomly determined from a uniform distribution in the range of 10% to 50% ([0.1, 0.5]) of the total area. This ensures that the dataset includes images with diverse filler loading levels. Filler agglomerates were modeled in the simulation as spherical structures. The radius r of individual particles was generated to follow an inverse power law. This is a distribution where the probability of a particle's appearance, $P(r)$, is inversely proportional to the square of its radius, such that $P\left( r \right)\propto r^{-2}$. This model was adopted because this distribution has scale-free characteristics and is widely observed in the particle size distributions of real materials and in natural phenomena. The radius of the generated particles was set to a minimum of 1 pixel and a maximum of 28.5 pixels. This maximum value was determined based on the largest agglomerate radius observed in the actual microscopy images (19 pixels), with a 1.5x margin applied. Based on the target coverage ratio and size distribution determined in the steps above, a list of particle radii to be drawn in a single image is generated. For each particle in the list, a random central coordinate (x, y) is selected to place it entirely within the image boundaries. Overlapping between particles is permitted to reproduce a more natural state of agglomeration. Each particle is drawn in a dark color similar to the fillers. The brightness value is 0 and it is used to fill the particle as a solid circle.

Figure S5. Ground Truth Image Generation Algorithm Flowchart.

**4. Knife-mark Noise Generation Algorithm**

This flowchart shows the procedure for generating knife-mark noise from a single Ground Truth image. First, in the Select Random Angle step, a single random angle (0-180°) is chosen to determine the direction of the noise for the entire image. Next, the Initialize Line Position step sets the starting position for drawing the lines at the outer edge of the image. A loop then begins, drawing lines one by one from one edge to the other until the image is covered. Inside the loop, the Select Random Line Parameters step randomly determines the color (brightness 0-100 in 8‑bit scale) and thickness (1-3 pixels) for each individual line. After a line is drawn at its calculated coordinates, the Update Next Line Position step moves the drawing position for the next line by a random spacing. This process is repeated until the drawing position moves beyond the edge of the image, ensuring it's completely covered. This generates a set of parallel lines with irregular spacing. Finally, this process outputs the Noisy Image, which is the original Ground Truth image with the noise superimposed on it. Note that during the actual dataset creation, a Line-only Image is also created by placing the same noise pattern on a blank white background.

Figure S6. Flowchart of the Knife-mark Noise Generation Algorithm.

**5. U-Net Training Details and Learning Curves**

Figure S7. Learning curves for the U-Net models. This figure compares the training progress of the two U-Net models used in this study. (a) The learning curve for the comparative U-Net model, which was trained on Gaussian noise. The stable convergence confirms that the model was trained successfully without significant overfitting. (b) The learning curve for the proposed U-Net model, which was trained on task-specific knife-mark noise. The validation loss closely tracks the training loss throughout the process, demonstrating robust learning and good generalization.

The same set of hyperparameters was used for training both the proposed U-Net (knife-mark noise) and the comparative U-Net (Gaussian noise) to ensure a fair comparison. The model was trained for 60 epochs using the Binary Cross-entropy loss function. The final layer of the network employs a sigmoid activation function to produce probability outputs. An Adam optimizer was utilized with an exponential decay learning rate schedule. Initial Learning Rate is 0.0001. Decay Schedule is the learning rate that was multiplied by a factor of 0.9 every 100 steps (staircase=True). The only difference between the two models was the training dataset used. To verify that both models were trained properly without significant overfitting, we tracked the loss (Binary Cross-Entropy) for both the training and validation sets at each epoch. As shown in Supplementary Figure S7, for both the knife-mark noise model and the Gaussian noise model, the training and validation loss curves converged smoothly while remaining in close proximity. This signifies that both models completed their training stably without succumbing to overfitting, thereby confirming that a foundation for a fair comparison of their performance has been established.

**6. Quantitative Comparison and Hyperparameters of Comparison Methods**

The hyperparameters for each comparison method were optimized in the section “Performance Evaluation of Noise Removal Based on Simulated Data” by minimizing the Mean Absolute Error (MAE) on a single representative pair of a simulated noisy image and its ground truth. The visual outcomes of this optimization are presented in Figure 2 of the main text, and the corresponding MAE values are shown in Supplementary Figure S8.

Below are the specific parameter settings used for each of the conventional comparison methods.

Median Filter: The filter was applied with a 5$\times$5 kernel (size: 5).

Band-stop Filter (FFT): A ring-shaped band-stop filter was applied in the frequency domain to remove components with radii between 46 and 130 pixels from the spectrum's center.

L1 Regularization: The reconstruction used a regularization coefficient (λ_L1_) of 1.90. The optimization was performed on a GPU with a learning rate of $1.0 \times{10}^{-4}$ and a convergence tolerance of $1.0 \times{10}^{-3}$.

TV Regularization: For TV reconstruction, the regularization coefficient (λ_TV_) was set to 1.00. The optimization parameters were identical to the L1 method (learning rate: $1.0 \times{10}^{-4}$, tolerance: $1.0 \times{10}^{-3}$) and executed on a GPU.

GSR (General Stripe Remover): The algorithm was run for 7000 iterations with projection enabled (proj: True). The regularization parameters (μ_1_,μ_2_) were set to 9 and 0.01, respectively. The stripe removal direction was fixed to horizontal (0 degrees).

Figure S8. Quantitative comparison of noise removal performance for each method using Mean Absolute Error (MAE). The MAE was calculated between the processed image and the ground truth image on the simulated dataset. Lower values indicate better performance. The gray dashed line represents the MAE of the original 'Knife-mark noise' without any denoising (baseline). The proposed U-Net (Knife-mark Noise) achieved the lowest MAE (3.27), outperforming all conventional methods and the U-Net trained on Gaussian noise. This result demonstrates the effectiveness of training with task-specific noise.

The hyperparameters for each comparison method were optimized in the section “Application to Real Images and Validation of Effectiveness” by minimizing the Mean Absolute Error (MAE) across 12 manually annotated real images. Figure 4 in the main text presents a representative example from this dataset, and Figure 5 shows violin plots summarizing the MAE distributions over all images. The corresponding processed results for the 12 cases are shown in Section 9 of the Supplementary Material. The parameter search results for the L1 and TV regularization methods are shown in Supplementary Figures S9 and S10, respectively.

Below are the specific parameter settings used for each of the conventional comparison methods.

Median Filter: The filter was applied with a 17$\times$17 kernel (size: 17).

Band-stop Filter (FFT): A ring-shaped band-stop filter was applied in the frequency domain to remove components with radii between 11 and 165 pixels from the spectrum's center.

L1 Regularization: The reconstruction used a regularization coefficient (λ_L1_) of 2.1. The optimization was performed on a GPU with a learning rate of $1.0 \times{10}^{-4}$ and a convergence tolerance of $1.0 \times{10}^{-3}$.

TV Regularization: For TV reconstruction, the regularization coefficient (λ_TV_) was set to 0.8. The optimization parameters were identical to the L1 method (learning rate: $1.0 \times{10}^{-4}$, tolerance: $1.0 \times{10}^{-3}$) and executed on a GPU.

GSR (General Stripe Remover): The algorithm was run for 7000 iterations with projection enabled (proj: True). The regularization parameters (μ_1_,μ_2_) were set to 9 and 0.01, respectively. The stripe removal direction was fixed to vertical (90 degrees).

Figure S9 Effect of L1 Regularization Parameter (λ_L1_) on Mean Absolute Error (MAE).

**A box plot showing the relationship between the regularization parameter λ_L1_ and the Mean Absolute Error (MAE) for image reconstruction using L1 regularization. The x-axis represents the λ value, varied from 0.1 to 5.0, while the y-axis shows the MAE. The MAE values represent the average over 12 manually annotated real images. The plot visualizes the distribution (median, quartiles, and mean) of MAE for each λ_L1_ value. The parameter search revealed that the minimum MAE was obtained at λ_L1_ = 2.1.**

Figure S10 Effect of TV Regularization Parameter (λ_TV_) on Mean Absolute Error (MAE).

**A box plot showing the relationship between the regularization parameter λ_TV_ and the Mean Absolute Error (MAE) for image reconstruction using TV regularization. The x-axis represents the λ value, varied from 0.1 to 2.1, while the y-axis shows the MAE. The MAE values represent the average over 12 manually annotated real images. The plot visualizes the distribution (median, quartiles, and mean) of MAE for each λ_TV_ value. The parameter search revealed that the minimum MAE was obtained at λ_TV_ = 0.8.**

**7. Analysis of GSR Parameter Sensitivity**

Figure S11. Effect of the angle parameter on GSR performance. The figure displays the results of applying the General Stripe Remover (GSR) to a real microscopy image while varying the angle hyperparameter from 50° to 125° in 5° increments.

The results clearly show that the method's performance is highly dependent on the input angle. Excellent removal of the knife-mark noise is achieved within a narrow optimal range of approximately 80° to 100°, which corresponds to the orientation of the artifacts in this specific image. Outside of this range, however, the method's effectiveness degrades significantly, failing to remove the noise. While GSR is remarkably effective at selectively removing this directional noise, it is designed to preserve other image features. Consequently, the fine background texture of the rubber material remains perfectly intact. For the specific task of filler extraction, where the goal is to remove all non-filler patterns (both knife marks and background texture), this selective nature is a limitation. This highlights that while GSR is a powerful tool for stripe removal, it may not be the most effective solution for this particular application.

**8. Local Variation of Noise Angles**

Figure S12. Example of complex stripe artifacts in a real-world image, illustrating the challenge for automated angle detection.

(Left) An original microscopy image displaying knife-mark artifacts. (Right) The same image with the artifacts' paths highlighted in red.

The lines show that real-world noise is not perfectly linear but can be curved and exhibit locally varying angles. This complexity makes it difficult to define a single, global removal angle, which is a necessary parameter for conventional methods like GSR, thus complicating fully automated processing.

9. **Summary of image processing results**

Figure S13. Comparison of image processing methods using the test images shown in Figure S1(a).

Figure S14. Comparison of image processing methods using the test images shown in Figure S1(b).

Figure S15. Comparison of image processing methods using the test images shown in Figure S1(c).

Figure S16. Comparison of image processing methods using the test images shown in Figure S1(d).

Figure S17. Comparison of image processing methods using the test images shown in Figure S1(e).

Figure S18. Comparison of image processing methods using the test images shown in Figure S1(f).

Figure S19. Comparison of image processing methods using the test images shown in Figure S2(g).

Figure S20. Comparison of image processing methods using the test images shown in Figure S2(h).

Figure S21. Comparison of image processing methods using the test images shown in Figure S2(i).

Figure S22. Comparison of image processing methods using the test images shown in Figure S2(j).

Figure S23. Comparison of image processing methods using the test images shown in Figure S2(k).

Figure S24. Comparison of image processing methods using the test images shown in Figure S2(l).
